# Hyaluronic Acid-modified Liposomes for Ursolic Acid-targeted Delivery Treat Lung Cancer Based on p53/ARTS-mediated Mitochondrial Apoptosis

**DOI:** 10.5812/ijpr-131758

**Published:** 2023-04-07

**Authors:** TingTing Ma, Jiasi Zhou, Jiajie Li, Qi Chen

**Affiliations:** 1 Department of Infectious Diseases, Ningbo Yinzhou No.2 Hospital, Ningbo, China; 2 Department of Respiratory and Critical Care Medicine, Ningbo Yinzhou No.2 Hospital, Ningbo, China; 3 The Affiliated Hospital of Medical School, University of Ningbo, Ningbo, China

**Keywords:** Ursolic Acid, Hyaluronic Acid, Liposome, Mitochondrial Apoptosis

## Abstract

**Background:**

Chemotherapy drugs can cause drug resistance and other problems when treating lung cancer, which leads to treatment failure. Ursolic acid (UA) is used in formulations based on traditional Chinese medicine. UA has excellent anti-tumor effects, but they are limited by solubility and non-specificity to tumor cells.

**Objectives:**

To overcome these issues, we created a novel hyaluronic acid (HA)-targeted liposome system for delivering UA (HA-Lipo/UA) to explore the targeting and anti-tumor effects of UA.

**Methods:**

We constructed the HA-Lipo/UA delivery system by the thin film hydration method. The uptake and localization of UA were detected by flow cytometry and microscope. Cell proliferation of A549 cells was detected by MTT assays. Apoptosis and reactive oxygen species (ROS) expression of A549 cells were also evaluated after being treated with HA-Lipo/UA. Western blot analysis evaluated the anti-tumor mechanism of HA-Lipo/UA.

**Results:**

HA-Lipo/UA exhibited favorable targeting of the cluster of differentiation (CD)44-overexpressing A549 cells. HA-Lipo/UA exhibited significant inhibition of the proliferation of A549 cells and induced their apoptosis compared with the corresponding monotherapies. HA-Lipo/UA induced overexpression of reactive oxygen species and upregulated expression of p53 and apoptosis-related protein in the transforming growth factor-β signaling (ARTS) pathway, which induced cytochrome-c release, activation of caspase-3, and promoted mitochondrial apoptosis in A549 cells.

**Conclusions:**

Taken together, these data suggested that HA-Lipo/UA could be used to target tumor cells.

## 1. Background

Lung cancer (LC) is one of the most commonly diagnosed cancers and the leading cause of cancer-related death worldwide (1). Approximately 2.2 million new cases are diagnosed worldwide, with ~1.76 million deaths, per year. Non-small-cell lung cancer (NSCLC) is the cause of > 80% of LC cases (2). The main challenges of NSCLC are that it is usually diagnosed at an advanced stage and that efficacious therapy is unavailable (3). First-line treatment for NSCLC is cisplatin or carboplatin-based doublet chemotherapy, but this treatment regimen can lead to severe nausea and vomiting, renal toxicity, and neuropathy (4). In addition, small-molecule inhibitors of epidermal growth factor receptor tyrosine kinases (e.g., erlotinib, gefitinib) are a second-line treatment for NSCLC (5, 6). Although chemotherapeutic regimens are highly efficacious for treating NSCLC, they can elicit multidrug resistance, side effects, and weak effects against metastasis and invasion, which leads to therapeutic failure (7). Hence, the discovery of safe and efficacious drugs for NSCLC is needed urgently.

Traditional Chinese medicine (TCM) formulations have been used in cancer therapy because of their few side effects and low drug resistance (8, 9). Ursolic acid (UA) is present in various TCM formulations, such as Forsythia and Loquat. UA has been demonstrated to have anti-tumor, antibacterial, and anti-inflammatory activities (10-12). Lin and colleagues reported that UA could treat oral squamous cell carcinoma by regulating protein kinase B/mammalian target of rapamycin/nuclear factor-kappa B (Akt/mTOR/NF-κB) signaling to induce apoptosis and autophagy in Ca922 cells (13). Despite these excellent anti-tumor effects of UA, it has low solubility and a lack of specific targets, which result in low bioavailability and side effects. Therefore, developing safe and efficacious strategies using UA is urgently required.

To increase the water solubility and targeting of drugs, the construction of nano drug delivery systems (e.g., liposomes, nanoparticles) is a rational approach (14). Emilli and colleagues reported that chitosan-modified poly (lactic acid) nanoparticles increased the absorption and the UA bioavailability. The latter can also be co-dissolved with lipids and encapsulated in liposome bilayers (15). These basal nano drug delivery systems can utilize tumors' enhanced permeability and retention (EPR) effect, which affords efficient drug accumulation at the tumor site. The cluster of differentiation (CD) 44, folic acid, and other receptors show high expression in tumor tissues and are targets for nano-delivery systems (16). Hyaluronic acid (HA) is generally used in tumor-targeted nanoparticle modification. HA can target CD44 receptors and prolong its presence in blood circulation. Poudel team reported dual stimuli-responsive UA-embedded nanophytoliposome are enzymatically cleaved to release UA in the acidic pH of the tumor microenvironment and showed good activity of anti-head and neck squamous cell carcinoma. However, they did not explore its anti-tumor mechanism (17). Kang team research has shown that UA induces cell cycle arrest, apoptosis, and anti-tumor angiogenesis. UA exhibits anticancer activities by inhibiting MMP2 and PD-L1 expression through EGFR/JAK2/STAT3 signaling (18). Therefore, the anti-lung cancer mechanism of ursolic acid nano preparation by inducing apoptosis remains to be further explored.

**Figure 1.**
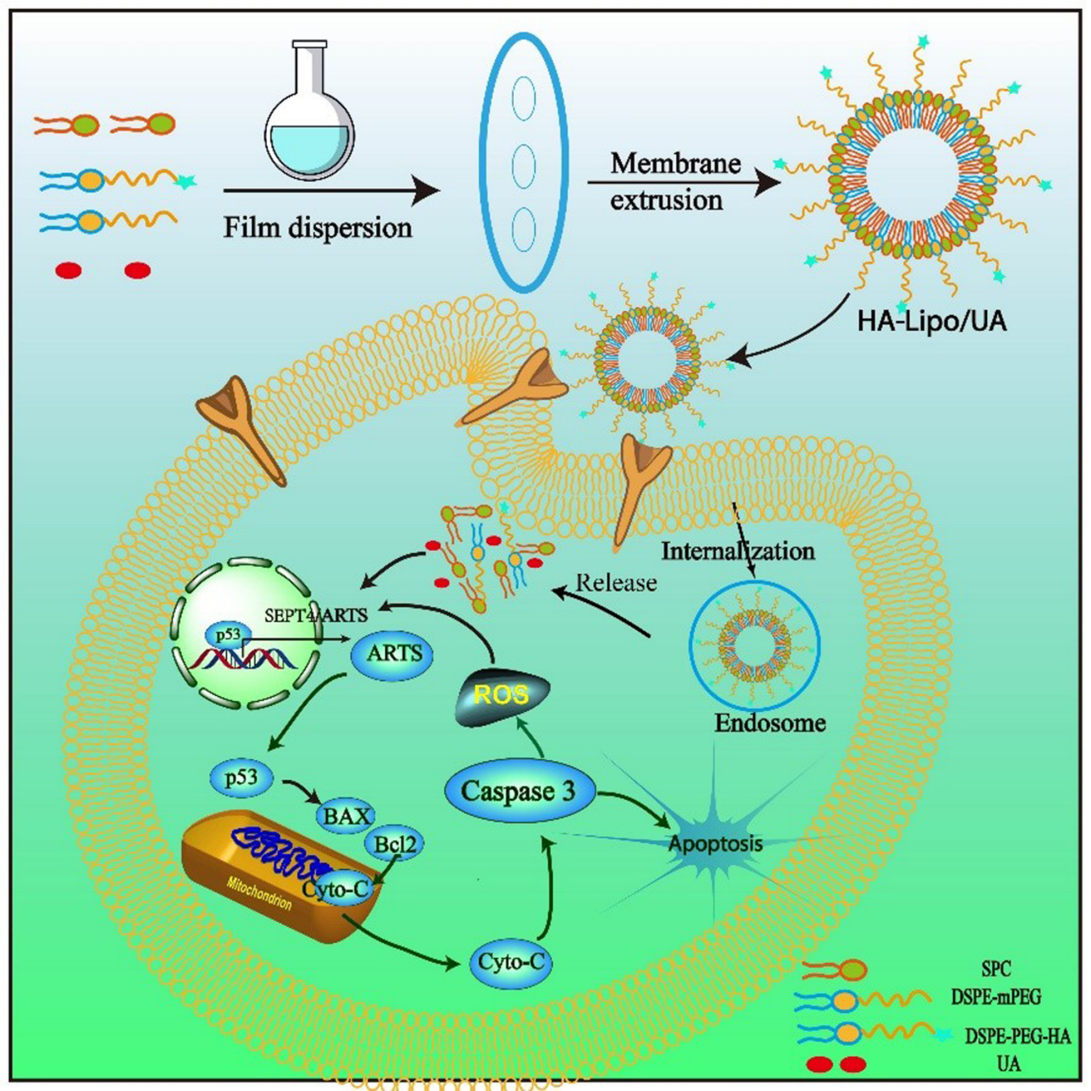
Schematic illustrations of the mechanism of HA-Lipo/UA inducing A549 cells apoptosis via p53/ARTS signal path.

In this study, to overcome the lipophilic and non-targeted of using UA in LC therapy, an HA-mediated nano-drug delivery system was designed (Figure 1). The lipophilic drug, UA, was embedded in an HA-modified liposome, which is a simple and easy preparation process. The enhanced bioavailability, sustained release, and increased tumor target of UA could be obtained by HA-modified liposome (HA-Lipo-UA). The targeting potential of the HA-Lipo-UA was investigated using in vitro studies. We investigated the anti-tumor activity of HA-Lipo-UA against A549 cells and the molecular mechanism related to mitochondrial apoptosis.

## 2. Objectives

Our study may aid the development of a productive strategy for tumor-targeting therapy.

## 3. Methods

### 3.1. Materials

Distearoyl-sn-Glycero-3-Phosphoethanolamine- PEG2000-Hyaluronan (DSPE-PEG2000-HA) was purchased from Ruixi Biological Technology (Xian, China). UA, lecithin, cholesterol, coumarin 6 (C6), a Mitochondrial Membrane Potential Assay Kit with JC-1, Reactive Oxygen Species Assay Kit, and Annexin V-FITC/PI Apoptosis detection kit were purchased from MeilunBio (Dalian, China). Antibodies against p53, ARTS, Bcl-2, Bax, Caspase 3, Glyceraldehyde-3-phosphate dehydrogenase (GAPDH) were purchased from Abcam (Baston, USA). A549 cells were purchased by m the Cell Bank of the Chinese Academy of Sciences (China).

### 3.2. HA-Lipo-UA Preparation

HA-modified liposomes were prepared as described previously (19). Liposomes consisting of lecithin, cholesterol, DSPE-PEG2000, and DSPE-PEG2000-HA (molar ratio = 1:1:0.1:0.1) were prepared using a thin-film hydration method. Briefly, UA and lipids (1:10, weight ratio) were dissolved in chloroform and then dried to a thin film by rotary evaporation (LC-RE-52A, Lichen, China) at 40°C. To ensure sufficient hydration, 5% glucose was added to the lipid film for 25 min at 45°C. Finally, liposomes underwent ultrasound using an ultrasonic processor (HENGMIN-R1200, Hengmin, China) for 5 min at 100 W at room temperature. The liposomes were extruded (Avanti mini extruder, Avanti, USA) through a polycarbonate membrane with 200 nm pores.

### 3.3. HA-Lipo-UA Characterization

The particle size and zeta potential of liposomes were measured at 25°C using a Zetasizer Nano instrument (Malvern Instruments, Worcestershire, UK). The morphology of liposomes was determined by transmission electron microscopy (Talos F200C, Thermo, USA).

The drug loading (DL) and EE of HA-lipo-UA were measured by ultrafiltration methods. In brief, liposomes were added to an ultrafiltration tube (10 kDa) to separate free UA after centrifugation (14,000 g, 20 min, 4°C). Then, vanillin (0.5%) in acetic acid solution and perchloric acid were added to the UA solution for 15 min at 60°C. Next, the absorbance of the solution was measured at 548 nm using a spectrophotometer (TU1905, Persee, China).

### 3.4. Stability of HA-Lipo/UA

The stability of HA-Lipo/UA was evaluated by dilution and in serum. Briefly, the liposome solution was diluted by phosphate-buffered saline (PBS, pH 7.4, 10 mM) at a ratio of 1:10, 1:50, and 1:250. To investigate the stability of liposomes in serum, HA-Lipo/UA was mixed with 10% fetal bovine serum 1:1 (v/v). Then, the size of liposomes was measured by Zetasizer Nano for stability analyses.

### 3.5. Drug Release In Vitro

The release profile of UA from liposomes was evaluated using a dialysis method, as described previously. Briefly, 1 mL of lipo/UA or HA-Lipo/UA was added to a dialysis bag with a molecular weight cutoff of 5000 Da. Then, the dialysis bag was soaked in release medium PBS at pH 7.4, and incubation was allowed at 37°C in a shaker. At this testing periods testing periods, the release medium (0.1 mL) was exchanged with the same volume of fresh medium. UA release was determined as described above.

### 3.6. Cellular Uptake

A549 cells were seeded in 24-well plates (5 × 10^5^/well). C6, Lipo/C6, and HA-Lipo/C6 were co-cultured with A549 cells for 4 h. Then, cells were fixed, and nuclei staining was done with 4′,6-diamidino-2-phenylindole at room temperature. After washing with PBS, fluorescence microscopy was undertaken to visualize the uptake of labeled liposomes by A549 cells. The cellular uptake of liposomes was also investigated by flow cytometry. C6 of different concentrations were co-cultured with A549 cells for 4 h. Then, cells were digested and analyzed by flow cytometry. Similarly, the kinetics of cellular uptake of HA-Lipo/C6 in A549 cells were analyzed using flow cytometry and fluorescence microscopy.

### 3.7. Cell Viability

The cytotoxicity of HA-Lipo/UA was determined using the 3-(4,5-Dimethylthiazol-2-yl)-2,5-diphenyltetrazolium bromide (MTT) assay. A549 cells were seeded into 96-well plates (8 × 10^3^ cells/well) and cultured overnight. They were treated with different concentrations of UA, Lipo/UA, or HA-Lipo/UA for 48 h. Then, cells were incubated with MTT for 4 h, and dimethyl sulfoxide (100 mL) was added to dissolve the crystals. Absorbance was measured with a microplate reader (K3, Thermo, USA) at 490 nm.

### 3.8. Apoptosis

A549 cells were seeded in 12-well plates (2 × 10^5^ cells/well) and cultured overnight. The cells were treated with UA, Lipo/UA, or HA-Lipo/UA (all at 20 µg/mL) for 24 h. Then, they were collected, washed, and digested. Next, they were stained using an Annexin V-FITC/PI Apoptosis Detection Kit (Becton Dickinson, Franklin Lakes, NJ, USA) according to manufacturer instructions and analyzed by flow cytometry. 

### 3.9. Assay to Measure ROS

A549 cells were seeded in 12-well plates (2 × 10^5^ cells/well) and cultured overnight. After treatment with UA, Lipo/UA, or HA-Lipo/UA (all at 20 µg/mL) for 24 h, the cells were incubated with a ROS kit for 30 min in the dark at 37°C. Intracellular ROS levels were analyzed by flow cytometry and fluorescence microscopy.

### 3.10. Western Blotting

A549 cells were seeded in six-well plates (5 × 10^5^ cells/well) and cultured overnight. After treatment with UA, Lipo/UA, or HA-Lipo/UA (all at 20 µg/mL) for 24 h, the cells were washed and lytic protein by RIPA buffer. The total protein was quantified using a BCA Protein Assay Kit. Proteins were separated by sodium dodecyl sulfate–polyacrylamide gel electrophoresis using 10% gels and transferred onto a polyvinylidene fluoride membrane. The membrane was blocked with 5% skim milk and then incubated with diluted primary antibodies (1:1000) in 5% skim milk with TBST at 4°C overnight. The membrane was washed with TBST and incubated with a secondary antibody. Then the proteins were imaged by a ChemiDoc™ XRS.

### 3.11. Statistical Analysis

The data were shown as mean ± SD. Statistical analysis was conducted using the GraphPad Prism software and performed by ANOVA. The Student's *t*-test was used to compare two groups, and P < 0.05 was considered statistically significant.

## 4. Results and Discussion

### 4.1. Preparation and Characterization of HA-Lipo/UA

Liposomes are often used as drug-delivery systems for treating various diseases because of their high solubility, biocompatibility, and specific targeting (20). Therefore, we constructed HA-modified liposomes to encapsulate UA by a film-dispersion method.

**Figure 2.**
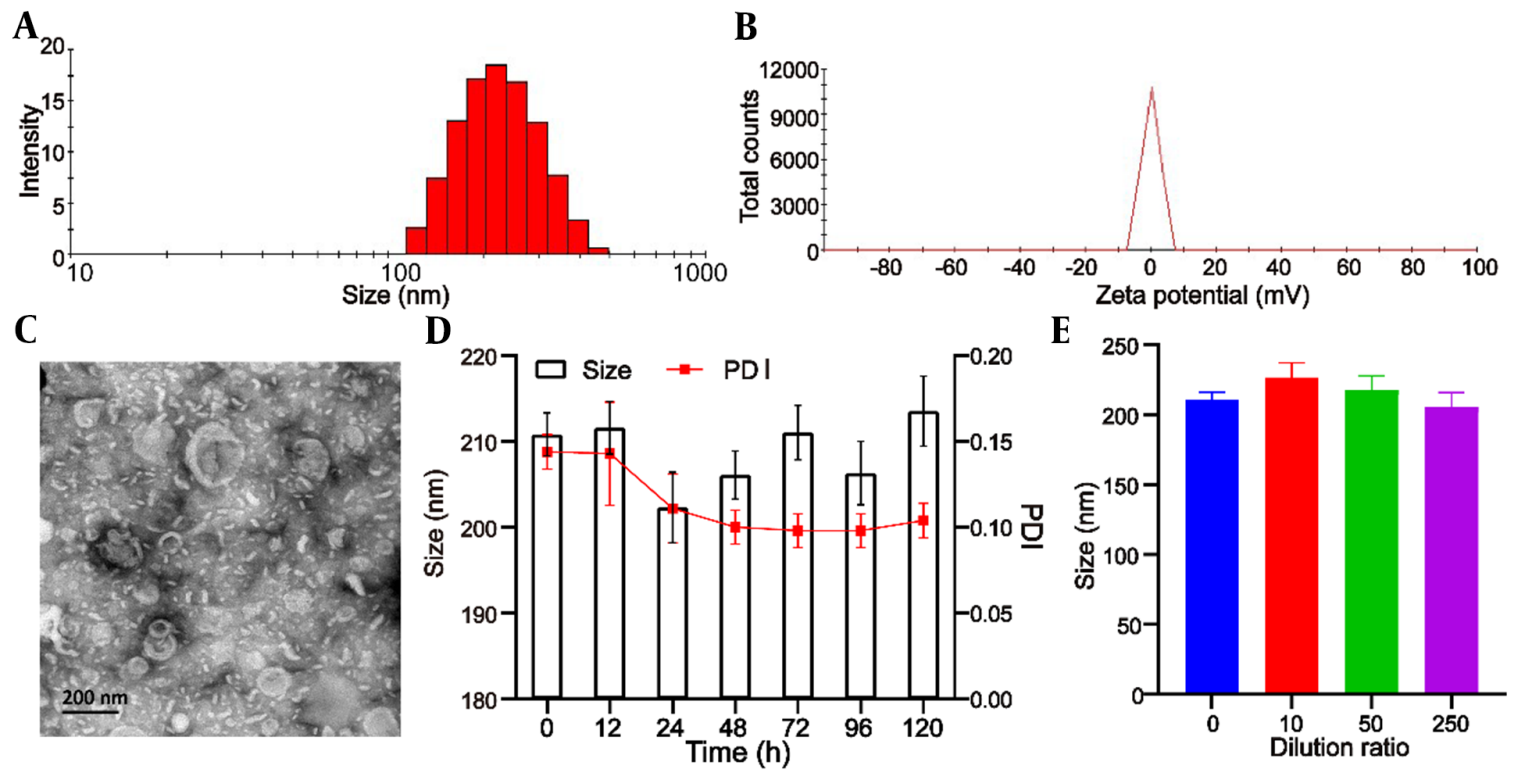
Characterization of HA-Lipo/UA. (A) Size distribution; (B) Zeta potential; (C) TEM image; (D) Serum stability; (E) Dilution stability.

The mean particle size of blank liposomes was 193.0 ± 5.3 nm, and the Polydispersity Index (PDI) was 0.193 ± 0.05. After encapsulation of UA, the mean particle size of Lipo/UA and HA-Lipo/UA was 205.1 ± 3.9 nm and 210.8 ± 4.5 nm (Table 1, Figure 2A) with a narrow PDI of 0.182 ± 0.03 and 0.144 ± 0.04, respectively, which demonstrated the uniform size of liposomes. The zeta potential of blank liposomes, Lipo/UA, and HA-Lipo/UA was −5.2 ± 0.3 mV, −4.3 ± 0.4 mV, and −13.5 ± 0.7 mV, respectively (Table 1, Figure 2B). The zeta potential of HA-Lipo/UA was lower than that of blank liposomes and Lipo/UA owing to the masked charge effect of PEGylation and HA having a negative charge. The EE of UA in Lipo/UA and HA-Lipo/UA was 88.9 ± 2.62% and 90.1 ± 2.43, respectively. The DL of UA in Lipo/UA and HA-Lipo/UA was 6.1 ± 0.21% and 5.9 ± 0.18%, respectively (Table 1). These results suggested that HA-Lipo could encapsulate UA efficiently. HA-Lipo/UA was spherical according to TEM, but the particle size was smaller than the detection limit of the particle-size analyzer (Figure 2C). These results indicated that liposomes of this size could increase drug accumulation at a tumor site by the EPR effect (21).

**Table 1.** The Characteristic of Nanoparticles

	Size (nm)	PDI	Zeta (mV)	EE (%)	DL (%)
**Blank-Lipo**	193.0 ± 5.3	0.193 ± 0.05	-5.2 ± 0.3	NS	NS
**UA-Lipo**	205.1 ± 3.9	0.182 ± 0.03	-4.3 ± 0.4	88.9 ± 2.62	6.1 ± 0.21
**HA-Lipo-UA**	210.8 ± 4.5	0.144 ± 0.04	-13.5 ± 0.7	90.1 ± 2.43	5.9 ± 0.18

The stability of liposomes is important for drug storage. We measured the particle size to illuminate the physical stability of liposomes. HA-Lipo/UA remained stable after incubation with PBS containing 10% fetal bovine serum for 120 h, and the particle size and PDI did not show obvious changes (Figure 2D). After HA-Lipo/UA had been diluted with different volumes of PBS, the particle size remained ~200 nm (Figure 2E). A negative charge and PEGylation have facilitated liposome stability (22, 23). These results suggested that HA-Lipo/UA had excellent stability for in vitro and in vivo research.

### 4.2. Drug Release In Vitro

**Figure 3.**
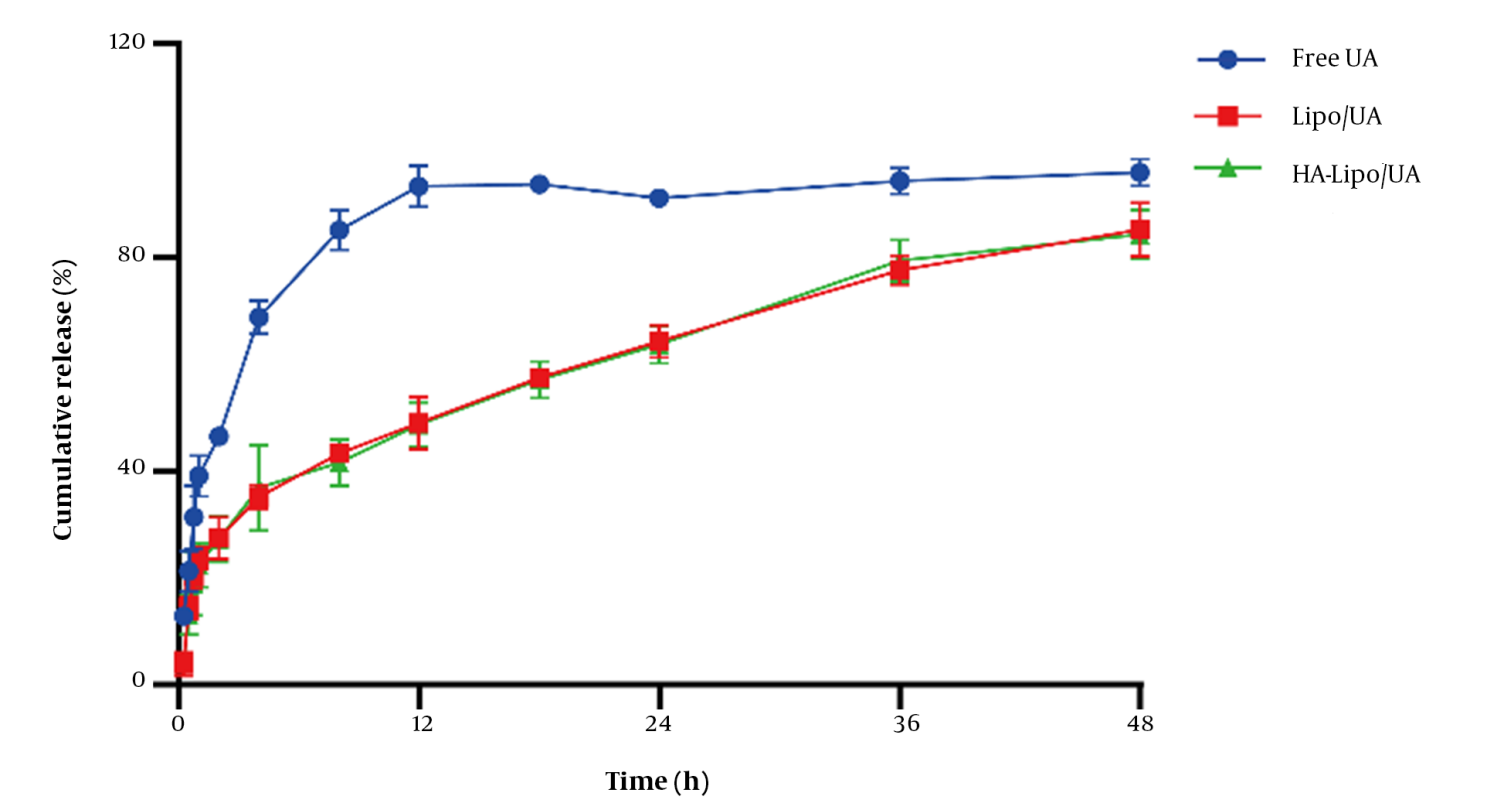
In vitro UA release of liposome in PBS buffer (pH 7.40).

UA release from liposomes in vitro was evaluated by a dialysis method. The amount of UA released from free-UA solution over 12 h was ~95% (Figure 3). Release of Lipo/UA and HA-Lipo/UA over 48 h was ~85%, with no obvious "burst release" at 30 min. These results showed that liposomes had significantly sustained release compared to the free-UA solution. The reason for the sustained release was that UA lies between phospholipid bilayers, which can reduce side effects and prolong the therapeutic effect of UA.

### 4.3. Cellular Uptake

**Figure 4.**
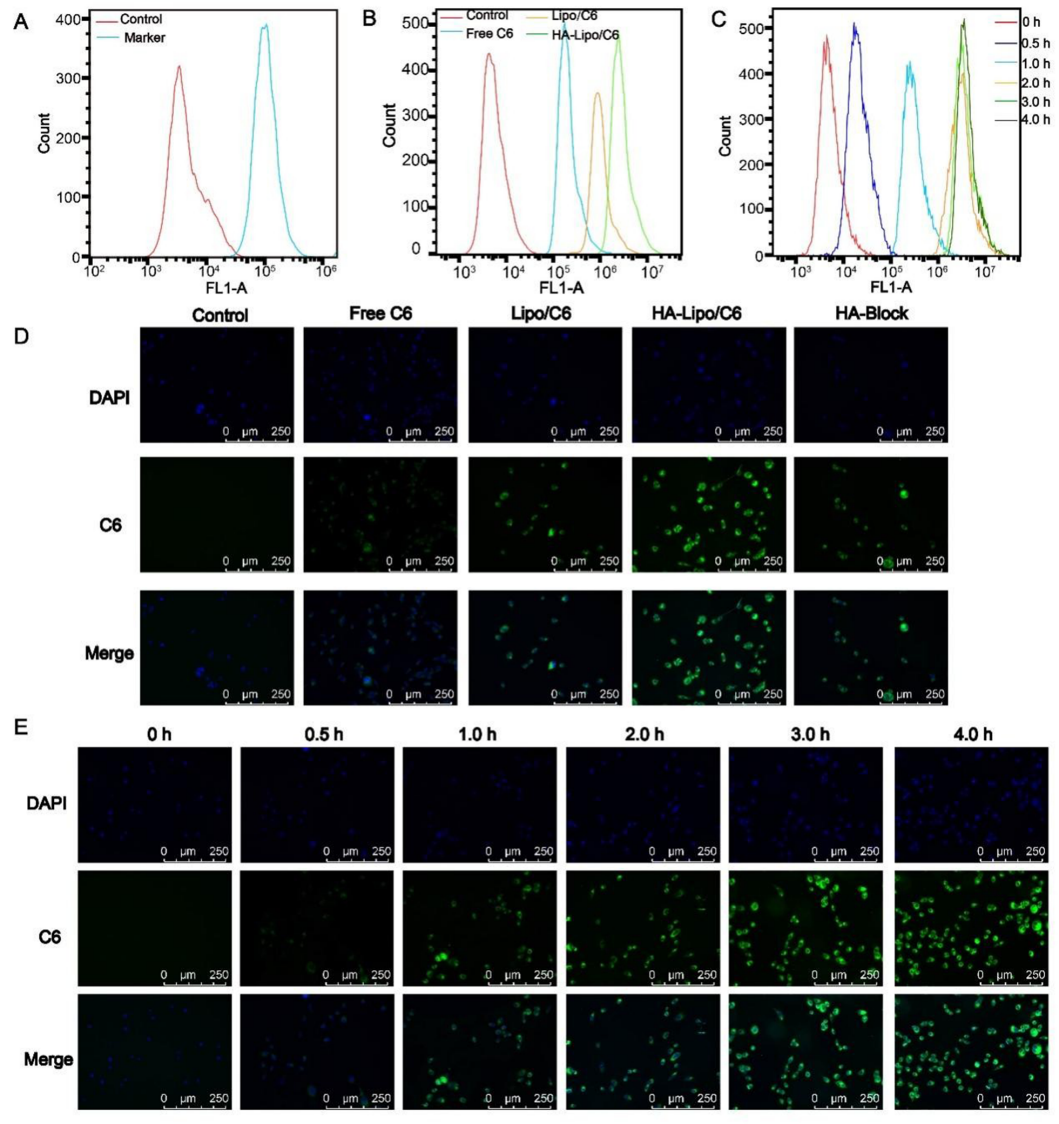
Cellular uptake of lipsome in the A549 cells. (A) CD 44 expression of A549 cells; (B) Cellular uptake of different formulation for 4 h by FCM; (C) Cellular uptake kinetics of HA-Lipo/UA at different time points by FCM; (D) Cellular uptake of different formulation for 4 h by fluorescence microscopic; (E) Cellular uptake kinetics of HA-Lipo/UA at different time points by fluorescence microscopic.

Cellular uptake was investigated using fluorescence microscopy and flow cytometry. To ensure the targeting of HA-Lipo/UA, we measured the expression of CD44 receptors on the surface of A549 cells: high expression was documented (Figure 4A) (24). Thus, A549 cells were selected for experiments targeting HA-Lipo/C6 uptake. Green fluorescence was detected in the cytoplasm of A549 cells, and the order of mean fluorescence intensity was HA-lipo/C6 > Lipo/C6 > free C6 (Figure 4D). We also used flow cytometry to quantify the mean fluorescence intensity. The mean fluorescence intensity of HA-lipo/C6 was higher than that of Lipo/C6 and free C6 according to flow cytometry (Figure 4B). This result was similar to that obtained using fluorescence microscopy. These results suggested that HA-Lipo/C6 delivered C6 into cells more efficiently than Lipo/C6 because of targeting of CD44 by HA in A549 cells. To determine the targeting effect of HA, researchers add HA to block CD44 receptors on the surface of A549 cells (25). The mean fluorescence intensity of HA-Lipo/C6 after HA's blockade of CD44 receptors was obviously reduced (Figure 4D). Thus, HA-Lipo/UA could effectively deliver UA to A549 cells by targeting CD44 receptors.

We also studied the kinetics of cellular uptake of HA-Lipo/C6. With increasing time, green fluorescence increased gradually, which indicated that cellular uptake increased in a time-dependent manner (Figure 4E). This result was consistent with analyses using flow cytometry (Figure 4C).

### 4.4. Cytotoxic Activity In Vitro

UA has excellent anti-tumor effects, and nanocarriers can be employed to increase these anti-tumor effects. The MTT assay was used to measure the anticancer activity of HA-Lipo/UA in vitro.

**Figure 5.**
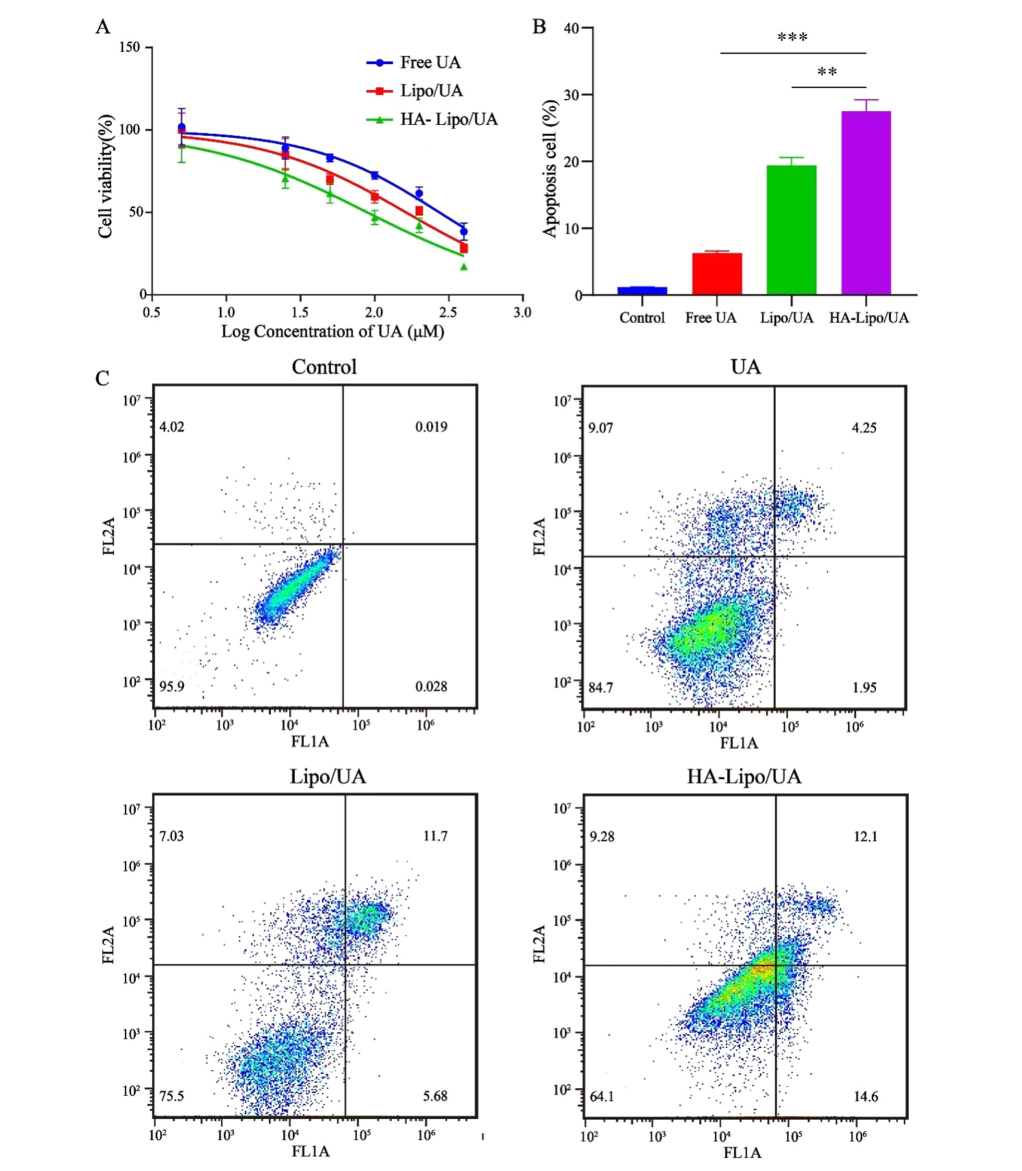
Antitumor activity assays. (A) Cell cytotoxicity test; (B) Quantification of the percentage of apoptosis; (C) FCM analysis of A549 cells with Annexin V-FITC and PI staining. *** P < 0.001, ** P < 0.01, and * P < 0.05.

The blank carrier was non-toxic with used concentrations by MTT assays (Appendix 1 in Supplementary File). Free UA, Lipo/UA, and HA-Lipo/UA (all at 5 - 400 µM) showed a high half-maximal inhibitory concentration (IC_50_) of A549 cells in a dose-dependent manner (Figure 5A). The inhibitory effects of UA samples were in the order HA-Lipo/UA > Lipo/UA > free UA with IC_50_ values of 89.37, 162.8, and 237.4 µM, respectively, after treatment for 48 h. These results suggested that HA-Lipo/UA displayed more potent inhibitory effects on tumor cells than those elicited by Lipo/UA and free UA, which may have been caused by greater drug uptake into cells and sustained release of HA-Lipo/UA (26).

### 4.5. Apoptosis Assay

Apoptosis is the main mode of cell death induced by anticancer drugs. Some researchers have suggested that UA can induce the apoptosis of hepatic carcinoma cells and breast cancer cells (27, 28). HA-Lipo/UA could strongly inhibit the proliferation of A549 cells in our study. Thus, we evaluated the apoptosis-inducing effect of HA-Lipo/UA (100 µM) on A549 cells using an Annexin-V-FITC/PI kit. Percent apoptosis of A549 cells after treatment with free UA or Lipo/UA was 6.3% and 19.4%, respectively (Figure 5B-C). These data suggested that liposomes, as drug carriers, could increase the percent apoptosis of A549 cells. Also, the highest percentage of apoptotic cells (27.5%) was noted with HA-Lipo/UA, followed by free UA and Lipo/UA. These results indicated that HA-modified liposomes increased the apoptosis of A549 cells by targeting them.

### 4.6. Assay to Measure Intracellular Levels of ROS

The high metabolic rate of tumor cells results in a higher intracellular ROS level than that in healthy cells (29). An anti-tumor strategy is to induce apoptosis by increasing ROS levels in tumor cells to inhibit the growth of tumor tissue (30). Rasat and colleagues suggested that UA disturbs ROS homeostasis and induces apoptosis in intestinal cancer (31). In our study, HA-Lipo/UA induced apoptosis, but its mechanism of action was not elucidated.

**Figure 6.**
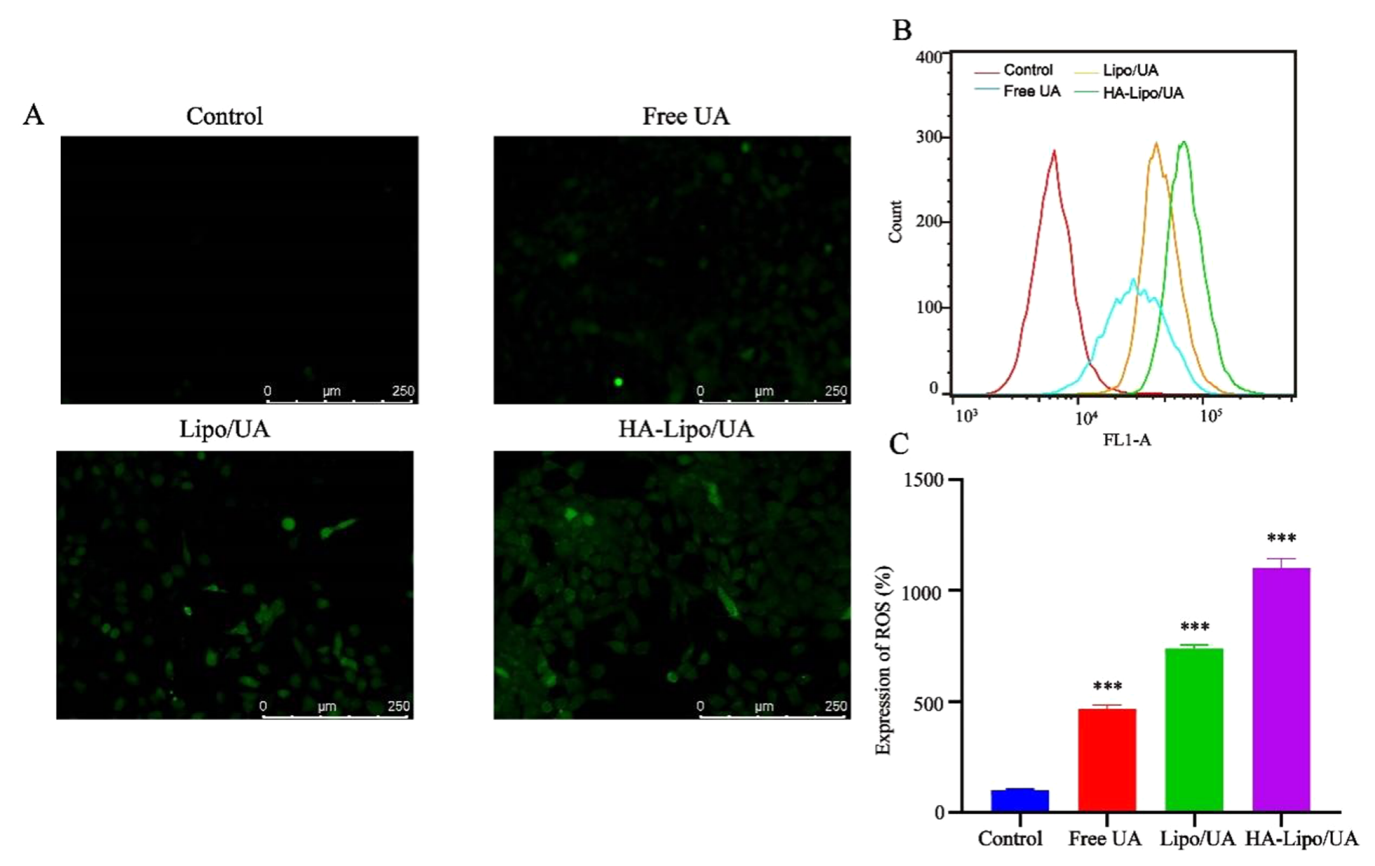
ROS expression in A549 cells after treat with different formulations. (A) Representative fluorescence images of ROS formation (DCFH-DA); (B-C) Flow analysis and quantification of ROS production. *** P < 0.001, ** P < 0.01, and * P < 0.05.

ROS were detected by the fluorescent probe 2′,7′-Dichlorofluorescin diacetate. The mean fluorescence intensity increased after treatment with UA, Lipo/UA, or HA-Lipo/UA (Figure 6A). In particular, HA-Lipo/UA showed stronger green fluorescence than Lipo/UA or free UA. Flow cytometry was employed to quantify the intracellular production of ROS. ROS levels in A549 cells after treatment with free UA, Lipo/UA, or HA-Lipo/UA (all at 100 µM) were 4.61-, 7.38-, and 11.0-fold higher than that of control cells, respectively (Figure 6B-C). Our results suggested that ROS were involved in the apoptosis of A549 cells induced by HA-Lipo/UA.

### 4.7. Effect of HA-Lipo/UA on Protein Expression of the P53/Apoptosis-related Protein in the ARTS Pathway

**Figure 7.**
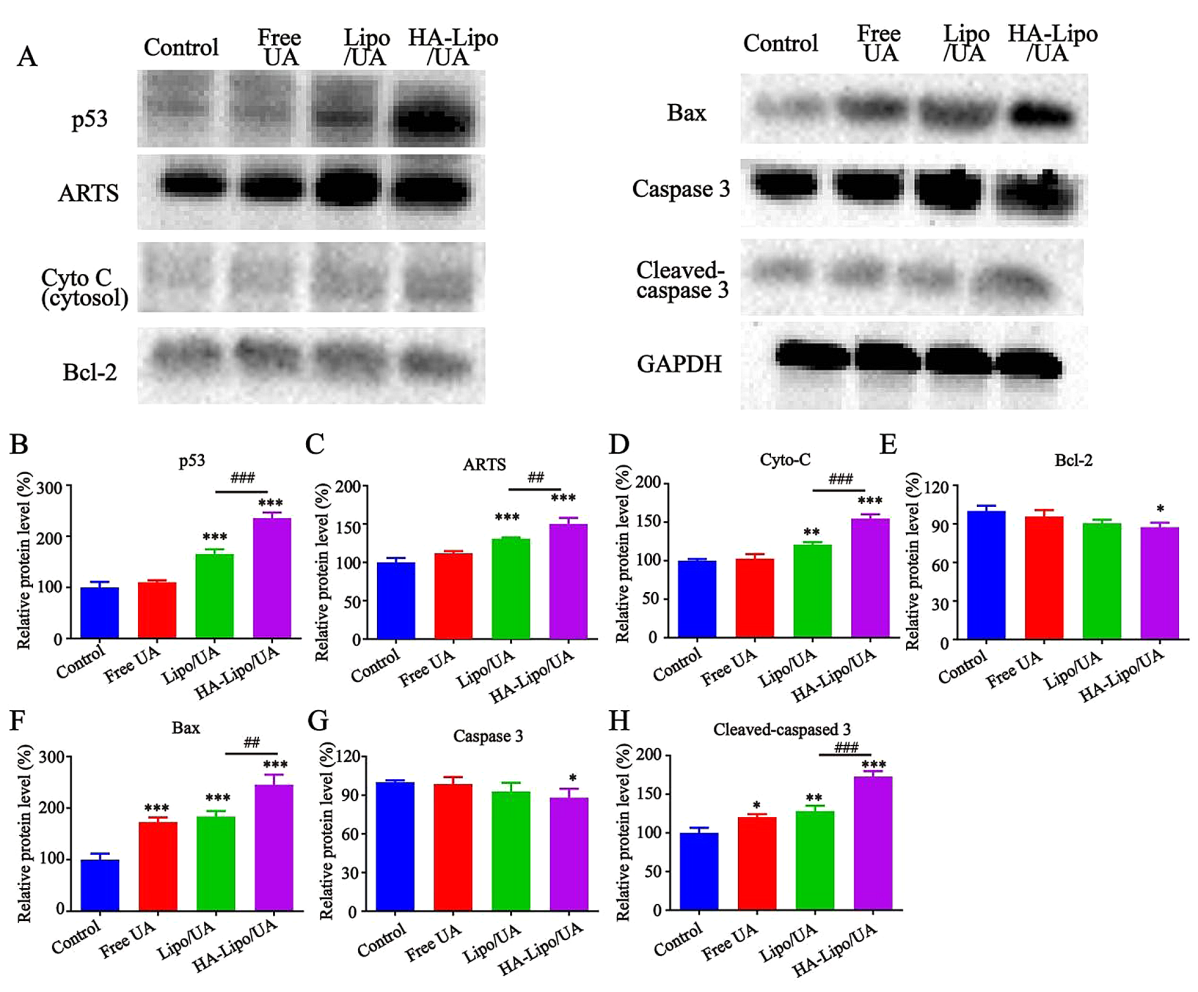
Effects of different formulations on p53/ARTS induce mitochondrial apoptotic signalling. p53, ARTS, Cyto C, Bcl-2, Bax, Caspase 3 and Gapdh protein expression in A549 cells detected by Western blot. Quantitative analysis of Western blotting bands including p53 (B), ARTS (C), Cyto C (D), Bcl-2 (E), Bax (F), Caspase 3(G) and Cleaved-caspased 3 (H). ***P < 0.001, ** P < 0.01, and * P < 0.05 compared to control group. ###P < 0.001 and ## P < 0.01 compared to Lipo/UA group.

In our study, HA-Lipo/UA could induce the apoptosis and increase the expression of ROS in A549 cells. Studies have shown ROS generation to be a significant factor in mitochondrial-dependent apoptosis (32). We wanted to investigate further the molecular mechanism by which HA-Lipo/UA influences A549 cells. Hence, we ascertained the effect of HA-Lipo/UA on the activation of various mitochondrial pathway-related apoptotic proteins by western blotting. After treatment with free UA, Lipo/UA, or HA-Lipo/UA, expression of p53, ARTS, Bax, cleaved-caspase 3 and cytochrome C was upregulated, and expression of Bcl-2 and caspase 3 was downregulated, in A549 cells (Figure 7A). HA-Lipo/UA had an obvious effect on protein regulation (Figure 7B–H).

p53 promotes cancer-cell death by activating apoptotic genes (e.g., Bcl-2 family proteins) (33). ARTS is a pro-apoptotic protein located at the outer membrane of mitochondria (34). Li and coworkers reported that ARTS could collaborate with p53 in mitochondria-engaged apoptosis. p53 can activate an apoptotic pathway by enhancing ROS accumulation, increasing Bax expression, and reducing protein expression of Bcl-2 (35)). It was found that HA-Lipo/UA promoted p53 activity and ARTS expression in A549 cells. By transcription, p53 induces expression of ARTS, which, in turn, interacts with and relocates p53 to mitochondria. The interaction between p53 with Bcl-2 can be enhanced by ARTS. This phenomenon was illustrated by HA-Lipo/UA inducing ROS overexpression and increasing interaction between p53 and ARTS to enhance mitochondrial apoptosis. Also, the balance between the expression of Bcl-2 and Bax is broken, which reduces the mitochondrial membrane potential and increases the release of cytochrome C from mitochondria to the cytosol. Finally, these proteins activate caspase-3 to promote apoptosis. Therefore, HA-Lipo/UA induced mitochondria-mediated apoptosis of A549 cells, which promoted cooperation between ARTS and p53, and increased ROS overexpression.

### 4.8. Conclusions

We demonstrated an effective system based on HA-Lipo/UA for treating A549 cells. HA-Lipo/UA exhibited excellent targeting of CD44 receptors and inhibited the proliferation of A549 cells thanks to enhanced UA accumulation in A549 cells via HA. In addition, HA-Lipo/UA upregulated the expression of ARTS and p53, and promoted mitochondrial apoptosis in A549 cells. Our drug-delivery system could be used to treat NSCLC.

ijpr-22-1-131758-s001.pdf

## Data Availability

The dataset presented in the study is available on request from the corresponding author during submission or after publication. The data are not publicly available due to the requirements of Laboratory.
