# Supplementary material for: Hyaluronic Acid-modified Liposomes for Ursolic Acid-targeted Delivery Treat Lung Cancer Based on p53/ARTS-mediated Mitochondrial Apoptosis
Source: Iran J Pharm Res. 2023 Apr 7;22(1):e131758. doi: 10.5812/ijpr-131758 (PMC10728842; doi:10.5812/ijpr-131758)
Supplement: ijpr-22-1-131758-s001.pdf [file ijpr-22-1-131758-s001.pdf]

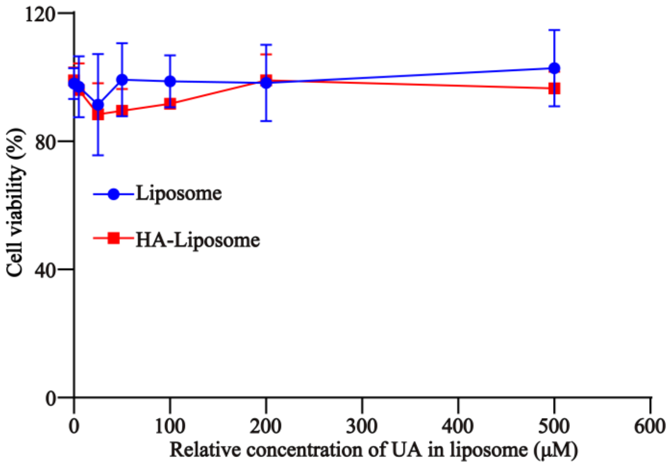

Appendix 1 Cell cytotoxicity test of blank carriers.

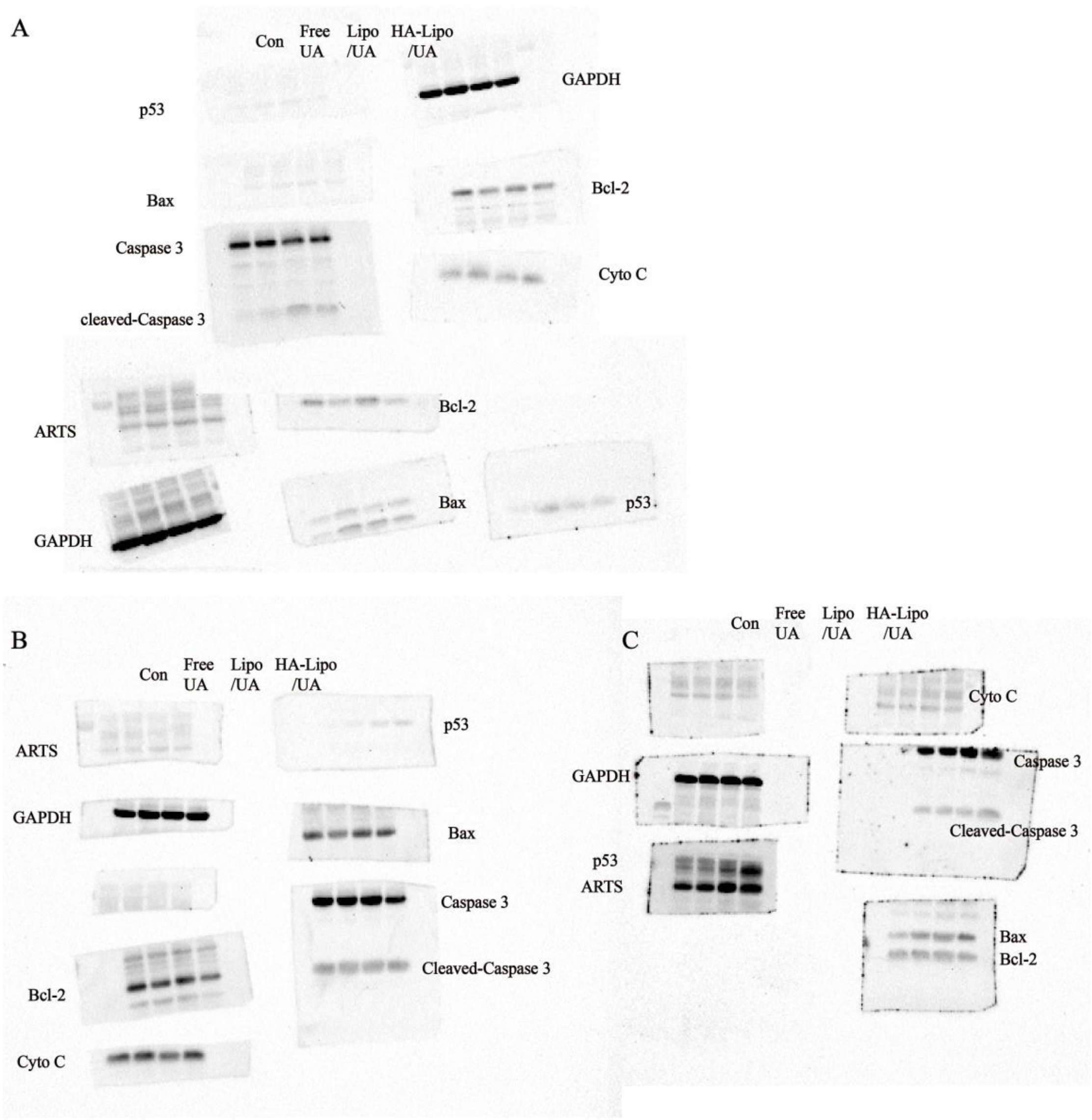

Appendix 2 Triplicate whole blot images. Channel labels are as following: con, free UA, Lipo/UA and HA-Lipo/UA (left to right).
